# Medical Students’ Efforts to Address COVID-19 Vaccine Hesitancy Through Motivational Interviewing

**DOI:** 10.7759/cureus.65755

**Published:** 2024-07-30

**Authors:** Karen Garza, Steven Latta, Cesar Larancuent, Kai Fu, Alexander Brown-Whalen, Jeffrey Eskra, Jiana T Baker, Silas Helbig, Jonathan Maya, Hani Samarah, Ronscardy Mondesir, Philip Desamour, Catherine Busatto, Shandi Brito, Prasad Bhoite, Frederick Anderson

**Affiliations:** 1 Department of Humanities, Health, and Society, Florida International University, Herbert Wertheim College of Medicine, Miami, USA; 2 Department of Medical Education, Florida International University, Herbert Wertheim College of Medicine, Miami, USA

**Keywords:** covid-19, neighborhoodhelp, south florida, public health education, community health, community outreach and engagement, medical school students, motivational interviewing, vaccine hesitancy, covid-19 vaccine hesitancy

## Abstract

Background

The coronavirus disease 2019 (COVID-19) pandemic reaffirmed health disparities in the United States (US) and highlighted the need for public health strategies to combat vaccine hesitancy, especially amongst vulnerable populations. The Green Family Foundation Neighborhood Health Education Learning Program (NeighborhoodHELP) at Florida International University (FIU) serves a predominantly uninsured population, making it a critical area of opportunity for addressing vaccine hesitancy. Motivational interviewing (MI), a technique that supports individuals in making autonomous health decisions, has shown promise in encouraging vaccine acceptance. Medical students at FIU’s Herbert Wertheim College of Medicine (HWCOM) are involved in the longitudinal care of the individuals in NeighborhoodHELP and receive training in MI within their clinical skills curriculum, making them optimally positioned to conduct outreach to encourage COVID-19 vaccination.

Project goals

There were two primary goals of this project: first, to systematically track and improve COVID-19 vaccination rates among individuals in NeighborhoodHELP, and second, to equip future physicians with hands-on experience in MI.

Methods

The COVID-19 Vaccination Promotion Initiative recruited medical students previously trained in MI to conduct outreach to unvaccinated individuals within NeighborhoodHELP. Students engaged in discussions about the COVID-19 vaccine with NeighborhoodHELP members, assisted in scheduling vaccination appointments, and updated medical records. The student team regularly met with faculty advisors to discuss changes in vaccine and public health data and to discuss challenges and successes with outreach efforts. To incentivize participation and enhance vaccine uptake, $25 gift cards were offered to individuals who agreed to receive the vaccine following the outreach conversations.

Results

From June 2021 to January 2023, the team made an estimated 720-1516 phone calls to NeighborhoodHELP individuals. The team encountered a challenge of low answering rates, with 35% of individuals being unreachable despite multiple attempts. Among those reached, 20% expressed no interest in receiving the vaccine, while 50% were interested in receiving the vaccine or had already been vaccinated. Vaccination rates among NeighborhoodHELP adults rose from 15.2% to 44.3% during this time.

Student experiences with MI were generally positive, with many noting success in engaging hesitant individuals. However, the team also encountered challenges, such as growing vaccine apathy within the community and difficulties in reaching patients via cold calls, which limited the overall impact of their outreach efforts.

Conclusions

By using MI techniques, medical students engaged with community members in meaningful conversations about the importance and safety of COVID-19 vaccination. However, the initiative fell short of the 50% vaccination target, facing challenges such as reliance on unsolicited phone calls and the complexities of incentivizing vaccinations through this outreach method.

Future initiatives could benefit from exploring alternative outreach methods, such as in-person engagement at community events or through partnerships with local organizations, to overcome the limitations of phone-based outreach. Additionally, investigating the relative efficacy of in-person versus telephone-based communication in promoting vaccination could provide valuable insights.

## Introduction

The coronavirus disease 2019 (COVID-19) pandemic had a devastating impact on public health in the United States (US) [1,2]. Robust clinical trials have repeatedly demonstrated that vaccination is a safe and effective method of preventing infections and reducing COVID-19 hospitalizations [3-5]. However, while the introduction of vaccines showed promise in the efforts to contain the virus, challenges such as breakthrough infections, viral mutations, and limitations in vaccine uptake threatened to attenuate their potential benefits. Several factors contribute to gaps in vaccine uptake across different demographic groups in the US, including barriers to vaccine access, widespread vaccine misinformation, and subsequent vaccine skepticism and hesitancy [6]. Addressing these barriers is essential to improving vaccination rates and protecting public health.

While there is conflicting research on vaccine hesitancy trends amongst different populations in the US, it is nevertheless clear that marginalized groups have experienced a disproportionately unfavorable impact on health outcomes as a result of the COVID-19 pandemic. This disparity reflects longstanding health inequities preceding the pandemic as well as the downstream effects of social and economic injustices that impact health and access to healthcare. Isasii et al. found that Indigenous, Black, and Latino individuals in the US were approximately three times more likely to be hospitalized and twice as likely to die from COVID-19 when compared to White, Asian American, and Pacific Islander individuals [7]. Additionally, marginalized communities experienced disparities in COVID-19 diagnostic testing, contact tracing programs, and resources for self-quarantine, all of which compound the burden of COVID-19 infection. 

Florida International University’s (FIU) Herbert Wertheim College of Medicine (HWCOM) provides healthcare services to a primarily uninsured population in the surrounding Miami-Dade community through the Green Family Foundation Neighborhood Health Education Learning Program (NeighborhoodHELP). Since 2010, this program has offered free preventive and primary healthcare to underserved households via mobile health centers (MHC), household visits, and, starting during the COVID-19 pandemic, telemedicine consults [8,9]. Most patients enrolled in NeighborhoodHELP identify as Black or Latino and face barriers to healthcare access, such as lack of insurance and financial constraints [8]. Considering the potential for increased COVID-19 burden and risk factors within the patient population, NeighborhoodHELP was a valuable avenue for promoting vaccine safety and efficacy to its community. 

We sought to improve COVID-19 vaccine uptake within the NeighborhoodHELP community by engaging in one-on-one discussions with members of the community who had not yet received the vaccine, using a technique known as motivational interviewing (MI). MI is a counseling approach that helps individuals find the self-driven motivation to make a positive behavior change, contrasting with more paternalistic health counseling methods. This client-centered approach is particularly effective for those who are ambivalent about changing their behaviors [10]. MI has been posited as a tool for combating vaccine hesitancy, as it supports autonomy while providing valuable information to individuals to guide them in their health decisions. This leads to a more informed decision about vaccination and greater levels of trust and cooperation between healthcare providers and community members [11]. 

Research shows that MI is efficacious in improving infant vaccination rates and rates of human papillomavirus vaccines [12,13], but its application in COVID-19 vaccine counseling is less explored. One randomized controlled trial found that MI increased COVID-19 vaccination uptake among intravenous drug users compared with a control group [14]. However, this study's small sample size (n=135) and specific patient population limit its generalizability. Considering the potential efficacy of MI and the centrality of its role in provider-patient counseling, this project offered opportunities for both improving vaccination rates in our community and providing an opportunity for FIU HWCOM medical students to hone their MI skills.

With this in mind, the COVID-19 Vaccination Promotion Initiative was designed to connect medical students trained in MI with unvaccinated NeighborhoodHELP community members. The goals of this project were to monitor vaccination rates among NeighborhoodHELP patients, increase vaccination rate (i.e., at least one shot) to beyond 50%, and provide medical students with experience in MI techniques.

The content of this paper was previously presented as a poster at the University of Miami Miller School of Medicine Department of Community Service (DOCS) Community Health Leadership Conference, which was held on April 21, 2023, in Miami, Florida, at the University of Miami Miller School of Medicine.

## Materials and methods

NeighborhoodHELP community

Eligibility to be contacted by the student team required NeighborhoodHELP individuals to meet specific inclusion and exclusion criteria. Inclusion criteria were: individuals must be at least 18 years old, and they must have had at least one clinic visit at the NeighborhoodHELP MHC after June 2019. Individuals with medical records indicating that they had already received the COVID-19 vaccine were excluded. Individuals were added to the contact list as they met eligibility criteria. Nine hundred and two out of all patients treated by the MHC (Table 1) met the inclusion and exclusion criteria by the end of the project.

**Table 1:** Demographic characteristics for adults enrolled in NeighborhoodHELP with at least one MHC visit after June 2019 NeighborhoodHELP: Neighborhood Health Education Learning Program; MHC: Mobile health center

Variables	Adults Enrolled in NeighborhoodHELP ( n=1184)
Gender, n (%)	
Female	768 (64.8%)
Male	416 (35.1%)
Preferred Language, n (%)	
English	504 (42.6%)
Spanish	503 (42.5%)
Haitian Creole	135 (11.4%)
Other/Unspecified	42 (3.5%)
Ethnicity, n (%)	
Hispanic or Latino	740 (62.5%)
Non-Hispanic or Latino	418 (35.3%)
Declined to Answer/Unspecified	26 (2.2%)
Race, n (%)	
White	665 (56.2%)
Black	397 (33.5%)
Asian	19 (1.6%)
American Indian or Alaska Native	4 (0.3%)
Declined to Answer/Unspecified	99 (8.4%)
Age, n (%)	
18-40 years	354 (29.9%)
41-64 years	640 (54.1%)
≥65 years	190 (16.0%)

MI training

MI is a longitudinal component of HWCOM’s curriculum for medical students. The technique is introduced at the end of the first year, during the advanced communication skills module of the clinical skills course. Over a period of three weeks, three to four hours per week are dedicated to the instruction and guided practice of MI, beginning with reflective listening techniques and subsequently introducing concepts of “change talk” and “sustain talk” (in which individuals use language that suggests a desire to either modify or maintain current behaviors, respectively). At the end of the module, students record themselves role-playing an MI scenario (e.g., involving tobacco cessation or lifestyle modifications) and receive individual feedback from faculty and peers. Training of MI continues in the second, third, and fourth years through student participation in NeighborhoodHELP. Students are assessed formally in MI skills through observed standardized clinical examinations (OSCE) at the end of their second year [15].

Phase I

In the summer of 2021, the HWCOM NeighborhoodHELP Quality Improvement Committee recruited 18 medical students from the FIU HWCOM class of 2024 to assist in this population health project. The FIU Institutional Review Board (IRB) approved the dissemination of de-identified data sets, analyses, and case studies developed for NeighborhoodHELP evaluation, program improvement, and required reporting to program funders (IRB-18-0039-CR03). Student volunteers were trained on the process for tracking calls and outcomes, as well as the procedure for updating patients’ vaccination status in the electronic medical record. These student volunteers had received training in MI techniques as described above, which provided a skillset for discussing the COVID-19 vaccine with patients. 

HWCOM faculty compiled lists of eligible, unvaccinated NeighborhoodHELP individuals quarterly and created spreadsheets to track call attempts and outcomes of those calls. The students contacted these individuals via unsolicited phone calls from the NeighborhoodHELP phone number. If the participants could be reached by phone, students introduced themselves, described their role within NeighborhoodHELP, and asked the individuals if they would be interested in talking about the COVID-19 vaccine. If individuals did not respond to the initial call, the attempt was documented, and the individual was contacted again, with a maximum of three attempts. Voicemails were not left, in an effort to protect privacy, given that many individuals’ phone numbers were shared with other household members. Individuals were contacted using their documented preferred language (including English, Spanish, and Haitian Creole) to reduce the limitations of language barriers, with professional translation services when needed. The students had access to a periodically updated document with answers to frequently asked questions about the vaccine, links to resources with vaccine information, local vaccination sites, and other resources compiled by the faculty team members. Students also had access to scripts and suggested conversation starters as further support for the MI technique. If NeighborhoodHELP participants were interested in receiving the vaccine, students could help guide them in making their appointments at local, free vaccination sites. Due to refrigeration requirements of the mRNA vaccines, the NeighborhoodHELP mobile health centers could not support storing these vaccines. For this reason, NeighborhoodHELP individuals interested in receiving the COVID-19 vaccine had to schedule appointments at outside vaccination sites. 

Periodic meetings between the students and faculty were conducted to review progress, discuss success stories and challenges, and develop mechanisms to improve the application of our initiative using the structure of Plan-Do-Study-Act cycles in Quality Improvement (QI) projects [16]. This approach led to discussions on reasons for hesitancy expressed by patients, how to approach circulating misinformation, and how students could be more adaptable during discussions with patients. These discussions also led to the integration of gift card incentives into the project.

Phase II

The initiation of the project's second phase was marked by a change in the student volunteer teams and the implementation of incentives for participants who received the COVID-19 vaccine after speaking to the team. Starting in June 2022, the group transitioned from primarily being composed of student volunteers from the FIU HWCOM Class of 2024 to students from the Class of 2025, to allow a new group of students to have an opportunity to engage with the project. Class of 2025 students also received curricular content on MI and aforementioned training on tracking the data and updating vaccination records within the electronic medical record system. All of the procedural aspects of the projects remained unchanged, aside from the implementation of incentives.

Incentives

In November 2021, the team secured an FIU-Baptist Health South Florida grant to incentivize COVID-19 vaccination through $25 gift cards for individuals aged 18-40 who received their vaccine series after speaking with the team. In September 2022, this grant was expanded to include booster shots, and in December 2022, it was further expanded to include all ages in our target population. To qualify for the gift card incentive, a NeighborhoodHELP individual had to speak to a student team member, agree to and attain a COVID-19 vaccination or booster, agree to a follow-up call from the team, and then consent to verification of their vaccine records in the Florida State Health Online Tracking System (SHOTS) database. Florida SHOTS is a free, statewide, centralized online immunization registry run by the Florida Department of Health. Once vaccination was confirmed, the participant would receive a gift card by mail.

Project conclusion

The vaccine promotion project was concluded in January 2023, following the announcement of the upcoming end of the COVID-19 pandemic national emergency in May 2023.

## Results

Calls and outcomes

Over the duration of this project, the student team made an estimated 720-1516 phone calls. The large range in the number of calls made is attributed to the varying frequencies with which individuals were contacted, ranging from one to three times per logged outcome. For instance, an individual who spoke with the team and agreed to get the vaccine might have been contacted several times to reach this outcome. The longitudinal nature of the project, spanning three years and involving multiple medical school classes and various spreadsheets, complicated accurate data tracking. However, our records indicate, confidently, that more than 720 calls were made. A significant and unforeseen barrier is the low answering rates. About 35% of the individuals identified for outreach were not able to be reached, comprising an estimated 366-524 calls (24%-73%). 

Of the 354 individuals who did answer the calls and speak with the student team, 70 individuals (20%) indicated that they were not interested in receiving the vaccine. According to the students, these calls varied greatly in content. Some individuals refused to discuss the matter with a stranger on the phone, while others were more willing to discuss their ambivalence and were open to having questions answered. Some even accepted a follow-up call in a few weeks to discuss the matter again.

Of the individuals who answered calls, 178 (50%) were documented as interested in receiving the vaccine or already vaccinated. In the earlier phases of the project, when the vaccine was new, these individuals often had questions and required assistance in finding vaccination sites. As the project progressed, the team encountered more individuals who were already vaccinated but may have still had questions about the appropriate time to receive a second vaccine or booster. As this project was initiated around the time when vaccines were first available, individuals with a single vaccine were considered “vaccinated” for the purposes of this project. 

The remaining 30% of successful contacts did not have a specific outcome logged due to data tracking inconsistencies amongst the large student group.

Throughout the project, NeighborhoodHELP individuals were periodically added to the spreadsheets for students to contact. At the end of the project, 228 individuals had not yet been contacted by the student team.

**Figure 1:**
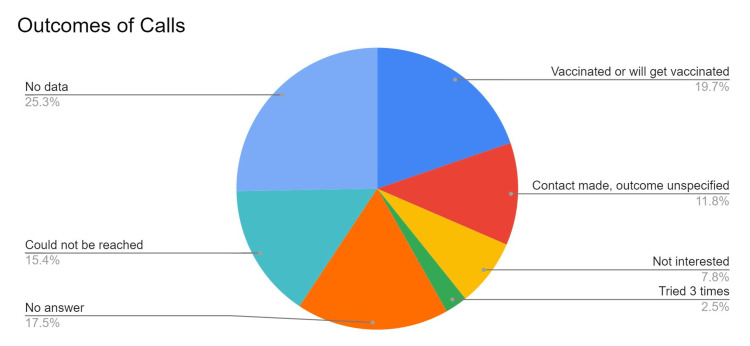
Outcomes of calls

The summary and visualization of call outcomes are shown in Table 2 and Figure 1 below.

**Table 2:** Call outcome descriptions NeighborhoodHELP: Neighborhood Health Education Learning Program

Call outcome	Description of call outcome	Instances logged
Will get vaccine or already vaccinated	Individuals who expressed interest in the vaccine or had already been vaccinated at least once.	178
Contact made, outcome unspecified	Individuals who were reached by the team, but for whom no specific outcome was recorded due to data collection inconsistencies.	106
Not interested	Individuals who indicated they were not interested in receiving the vaccine. Some individuals were willing to discuss their reasons for hesitancy and were invited to reach out to NeighborhoodHELP should they want to discuss the vaccines further. Some individuals accepted a follow-up call at a later time.	70
Tried three times	Individuals who did not answer after three attempts (the predetermined limit of attempts).	23
No answer	Individuals who did not answer after one or two attempts, but had yet to be attempted the maximum number of times.	158
Could not be reached	Individuals who could not be reached because they were no longer in NeighborhoodHELP or because their phone numbers were not in service.	139
No data	Individuals for whom no calls/outcomes were recorded, or who had not yet been contacted by the team by the end of the project.	228
TOTAL		902

Vaccination rates

When the project started in June 2021, out of the 873 adults enrolled in the NeighborhoodHELP who had recently received clinical care from the MHC, 133 (15.2%) had received at least one COVID-19 vaccine. By January 2022, this population grew to 958 participants, and the number of vaccinated individuals increased to 395 (41.2%), nearly tripling. By January 2023, 524 (44.3%) of the 1,184 adults had received at least one COVID-19 vaccine.

Table 3 demonstrates these described changes in vaccination status.

**Table 3:** Vaccination status of adults enrolled in NeighborhoodHELP at various points during the vaccine promotion project NeighborhoodHELP: Neighborhood Health Education Learning Program; MHC: Mobile health center

Time	Adults Enrolled in NeighborhoodHELP and seen at MHC within the last year (n=1184)	Adults Vaccinated	Percentage Vaccinated
June 2021	873	133	15.2%
September 2021	930	318	34.2%
January 2022	958	395	41.2%
May 2022	1011	416	41.1%
January 2023	1184	524	44.3%

Incentive gift cards

Ten gift cards of $25 each were given to individuals who received a COVID-19 vaccine or booster after speaking with the team.

Student experiences with MI

The regular meetings between the student team and faculty mentors allowed for constructive conversations about the successes and challenges of the project. The student team often shared positive experiences they had engaging with the NeighborhoodHELP community. The team often found that individuals appreciated opportunities to discuss the vaccines candidly, whether they intended to receive vaccinations or not. Moreover, students felt that this project was helpful practice in MI, particularly when coupled with the challenge of cold calling individuals to discuss a sensitive subject. The opportunity to practice MI with feedback from faculty mentors and support from peers was an invaluable extracurricular experience for the students. 

Some of the challenges faced by the team included increasing apathy toward the vaccines by community members over time and difficulty reaching patients via unplanned phone calls. Perhaps the most challenging obstacle was the very high frequency of unanswered outgoing calls reported by students, which was discouraging and time-consuming and made it difficult to reach all of the eligible NeighborhoodHELP individuals. 

Table 4 includes some examples of student perspectives taken from various points in time during the project. 

**Table 4:** Student comments about the experience with MI during outreach MI: Motivational interviewing

Selected Student Comments
“I spoke to someone who was very hesitant about the emergency use authorization and felt that the vaccine was made too quickly. I explained mRNA technology, that it has been in the works for many years, and that COVID was just an opportunity to put it into action. When I called them to follow up, they were less hesitant and eventually got the COVID vaccine.” -Brooke, Class of 2024
“Most people don’t want to feel pressured. When you use MI skills, you can find out why people are hesitant. They will often go do research on their own and when you call back a second time to follow up, they are more inclined to get the vaccine.” -Max, Class of 2024
“It’s been a growing experience to do the calls. It does feel like you’re bothering people sometimes, especially when they are very against the vaccine. You can give them more information if they want it, or ask a question to get a better understanding of their mindset. Cat Busatto told us that we are here to reach those on the fence, to help them come to the other side. It’s sad seeing people not getting the vaccine because of misinformation. A lot of the hesitant patients are from African-American backgrounds.” -Valandrea, Class of 2024
“This experience has given me another opportunity to engage with the NeighborhoodHELP patient community and to apply the motivational interviewing we learned in clinical skills. I have been surprised by the difference this patient-centered approach can have in changing the dialogue with vaccine-hesitant patients.” -Jeff, Class of 2025
“Working with the vaccine hesitancy project has been an insightful and rewarding way to contribute to the ongoing work toward mitigating the impacts of the COVID pandemic. Speaking with individuals about the vaccine has helped me to better understand common perspectives, concerns, and reservations that people hold regarding vaccines and the healthcare system generally. Engaging in these conversations has been a rewarding way to help support community health while strengthening my MI skills.” -Alex, Class of 2025

## Discussion

We found that throughout our initiative (June 2021 to January 2023), the number of patients who received vaccinations in the Miami-Dade community served by the NeighborhoodHELP increased from 15.3% in June 2021 to 44.3% in January 2023. While we did not reach our initial goal of 50%, this nearly three-fold increase suggests that our initiative may have contributed to a reduction in vaccine hesitancy amongst the program’s participants. However, it is difficult to isolate this project's impact from other initiatives at the broader county and state levels. 

**Figure 2:**
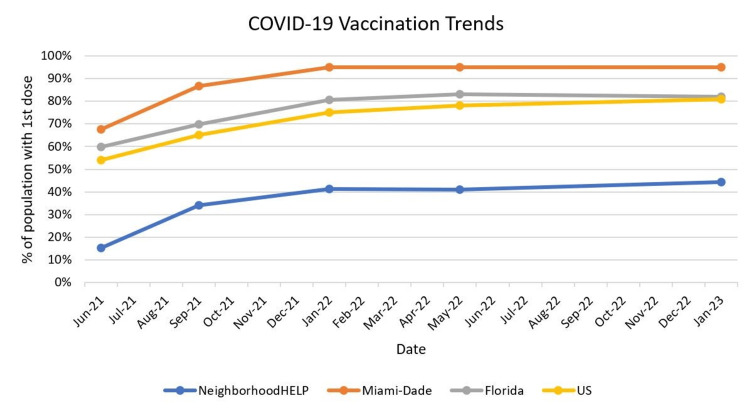
Vaccination rates in NeighborhoodHELP, Miami-Dade, Florida, and the United States NeighborhoodHELP: Neighborhood Health Education Learning Program

While the gap in the rate of vaccination in the NeighborhoodHELP community as compared to the general population was never closed, our project may have contributed to the increased uptake of the COVID-19 vaccine such that the upward trends stayed on par with the rise in vaccine uptake in the general Miami-Dade, Florida, and US populations. As shown in Figure 2, the CDC data suggested a 22.7% increase among Floridians and a 27.51% increase among Miami-Dade County residents throughout this timeline, compared to a 29.1% increase in the NeighborhoodHELP population. 

Figure 2 shows the changing vaccination rates in the NeighborhoodHELP population compared to the populations of Miami-Dade, Florida, and the US.

Reasons we believe the vaccination uptake rate slowed in 2022 and 2023 are vaccine fatigue, pandemic fatigue, misinformation, and politicization of vaccination, among others. While the initial cohort of students that worked on this initiative (Phase I) might have encountered more NeighborhoodHELP participants with interest in the newly available COVID-19 vaccines, the second cohort of students (Phase II) reported that most participants were uninterested in discussing the vaccine, as those who were willing to receive the vaccine likely would have done so by mid-late 2022.

Limitations

The most significant limitations of our outreach were the constraints caused by using unsolicited phone calls as the outreach method. Cold calling was time-intensive for the student team, particularly with the high rate of unanswered calls. Phone calls rather than in-person counseling might have also limited the development of rapport between the team members and patients. Patients were frequently skeptical about discussing their health with an unfamiliar caller. Furthermore, without a clinic to give the vaccines and monetary incentives, we relied on patients to schedule appointments themselves at local pharmacies. This created a challenge in both encouraging patients to agree to vaccination and then confirming that they had received the vaccine. Access to a clinic or space to give the vaccines ourselves could have significantly simplified the process for patients and our team. Further challenges included limited volunteer capacity and decentralized vaccine records. Vaccination discourse exhaustion and perceptions of vaccine irrelevance were frequently encountered obstacles, particularly in the later months of the project.

An additional limitation in our analysis is the lack of a control group. Since we did not establish such a group (i.e., matched individuals within the NeighborhoodHELP population who were excluded from our targeted outreach), it would not be possible to confidently ascertain the impact of our outreach efforts in improving vaccination rates in this population.

Our intention to incentivize vaccinations with gift cards was limited by the cumbersome process involved in the gift card disbursement and communication via unsolicited phone calls. To attain a gift card, an individual would have to answer the cold call, agree to receive the vaccination on their own, agree to receive another call to verify they had received the vaccine, allow permission for our team to access their Florida SHOTS record, and then provide us their mailing address. The logistical hurdles of accessing Florida SHOTS and the complex workflow to send the gift cards likely prevented the success of this incentive. This is especially evident by the lack of change in vaccination rates after the gift cards were introduced in November 2021. Several state programs have enrolled incentive programs, ranging from small and guaranteed rewards to lotteries for large prizes, to improve COVID-19 vaccination rates [17]. The effectiveness of such vaccination programs still needs to be determined. In a 2022 study on 24 statewide incentive programs and their data, incentive programs were not found to be associated with a significant change in COVID-19 vaccination rates but might have had small effects on vaccine uptake [18].

Future directions

As the cold calling method was one of the most limiting constraints of this initiative, future initiatives might benefit from a “hotline” approach instead. With this approach, community members could talk to a student volunteer about up-to-date COVID-19 vaccine information, access assistance scheduling a vaccine appointment, and receive their incentive after vaccination, at their own convenience. This method would bypass the time-intensive process of cold calling and may allow community members to seek information during their free time. This approach might have been particularly useful in the early months of vaccine availability when individuals had many questions and faced challenges in accessing vaccination appointments. We believe that this approach might have been popular amongst our community members, especially during the earlier months of the project, when confusion and interest about the newly released vaccines were highest. Even in some of the later months of the project, when people might have had more questions about the appropriate time to receive boosters, a hotline might have been a useful resource for NeighborhoodHELP community members. 

This initiative might have also benefited from working more directly with the MHCs. Providers at the MHCs might have been able to identify patients who indicated interest or ambivalence in the vaccine and connected these individuals with the student team, who could discuss the vaccine and assist in scheduling vaccine appointments. Alternatively, members of the student team could have been physically present at the MHCs in shifts to discuss vaccinations with MHC patients in person, which might have circumvented some of the difficulties in cold calling and establishing rapport over the phone.

Future efforts could explore the difference in efficacy of communicating in-person vs. via phone call during MI to determine the overall efficacy of telephone-based approaches. This could be of particular interest in cases in which no prior relationship exists between the caller and the patient contacted.

## Conclusions

The COVID-19 Vaccination Promotion Initiative aimed to address the critical issue of vaccine hesitancy within the NeighborhoodHELP community served by FIU HWCOM. Through a targeted approach utilizing MI techniques, medical students engaged with community members to discuss the importance and safety of COVID-19 vaccination. Over the course of our initiative, from June 2021 to January 2023, the vaccination rate among NeighborhoodHELP participants rose from 15.2% to 44.3%. While we fell short of our initial goal of achieving a 50% vaccination rate, this substantial increase suggests that our efforts might have played a role in reducing vaccine hesitancy. Our project also importantly provided an invaluable experience for medical students in applying MI skills, providing public health education, and engaging with underserved populations, especially discussing sensitive matters. However, our project encountered limitations, including the reliance on unsolicited phone calls for outreach and the complexity of incentivizing vaccinations through this challenging communication method. Moving forward, future initiatives could explore alternative outreach methods such as hotlines and closer collaboration with mobile health centers to overcome these challenges. Additionally, further research could investigate the efficacy of in-person versus telephone-based communication in motivational interviewing for vaccine promotion.

The COVID-19 Vaccination Promotion Initiative underscores the importance and challenges of community engagement and addressing vaccine hesitancy. By learning from our experiences, adopting improved outreach strategies, and continuing to foster collaboration between healthcare providers and underserved populations, we can continue to bridge the gap in vaccine hesitancy beyond the COVID-19 pandemic.
